# Depth-Dependent Performance of Residual Networks for Low-Count PET Image Restoration Using a Dedicated 3D-Printed Striatum Phantom

**DOI:** 10.3390/bioengineering13040392

**Published:** 2026-03-27

**Authors:** Chanrok Park, Min-Gwan Lee, Sun Young Chae

**Affiliations:** 1 Department of Radiological Science, College of Health Science, Eulji University, 553, Sanseong-daero, Sujeong-gu, Seongnam-si 13135, Republic of Korea; mglee2@g.eulji.ac.kr; 2 Department of Nuclear Medicine, Uijeongbu Eulji Medical Center, Eulji University School of Medicine, 712, Dongil-ro, Uijeongbu-si 11759, Republic of Korea

**Keywords:** low-count PET imaging, residual networks (ResNet), conventional filtering techniques, image restoration, 3D-printed phantom

## Abstract

Low-count positron emission tomography (PET) is inherently affected by Poisson-dominated noise, which degrades image contrast, structural delineation, and quantitative reliability. This study systematically evaluated residual learning-based deep neural networks to investigate the influence of residual block depth on PET image restoration performance under low-count conditions. We employed a physically controlled striatum phantom, fabricated using 3D printing technology, to ensure reproducible acquisition conditions and controlled physical variability. PET images were acquired using a clinical PET/computed tomography (CT) system with list-mode acquisition. Low-count images reconstructed from short-duration acquisition were paired with high-count reference images reconstructed from extended acquisitions. We compared conventional filtering techniques, including median, Wiener, and modified median Wiener filters, with residual network (ResNet)-based models incorporating 8, 16, and 32 residual blocks. Image quality was quantitatively assessed using contrast-to-noise ratio (CNR), coefficient of variation (COV), line profile analysis, universal quality index (UQI), and perceptual image patch similarity (LPIPS). The results demonstrated that ResNet-based restorations substantially outperformed conventional filtering techniques in contrast recovery, signal stability, and structural preservation. The ResNet-16 model achieved the most balanced performance, yielding the highest CNR (9.02) and lowest COV (0.105), while also demonstrating superior structural and perceptual similarity, as indicated by UQI (0.9224) and LPIPS (0.0174), relative to the high-count reference images. Deeper network configurations exhibited diminishing returns and reduced structural consistencies. These findings indicate that an intermediate residual block depth is optimal for low-count PET image restoration and highlight the importance of architectural optimization in deep learning-based PET image enhancement with phantom-based evaluation frameworks.

## 1. Introduction

Brain positron emission tomography (PET) is a molecular imaging modality that provides in vivo quantitative information on physiological and biochemical processes by detecting coincidence gamma photons emitted from positron annihilation [1,2,3]. In contrast to structural imaging modalities, such as computed tomography (CT) and magnetic resonance imaging (MRI), which primarily depict anatomical morphology, PET enables the visualization and quantification of tracer kinetics and regional radiotracer distribution, thereby reflecting underlying biological function. In PET imaging, image quality is governed by the number of detected coincidence events, which can be increased by administering higher radiotracer activity or extending the acquisition duration [4]. While higher injected activity improves counting statistics and the signal-to-noise ratio, it unavoidably increases patient radiation exposure. Extending the acquisition duration can partially compensate for low counts; however, prolonged scans increase patient burden and susceptibility to motion artifacts, and the relative contribution of random and scattered coincidences, which can degrade image quality [5,6]. Watson et al. reported that while increasing activity or duration improves the counting statistics and the peak noise equivalent count rate in PET imaging, these benefits are limited by radiation dose and by increased random and scattered coincidences [5]. Chang et al. further demonstrated that such improvements are highly patient-dependent, as body habitus and scanner characteristics significantly influence image noise, limiting the effectiveness of dose or time escalation, particularly in patients with a higher body mass index [6]. PET image formation relies on the stochastic detection of annihilation photon pairs, where the number of detected events follows Poisson counting behavior statistics, with variance proportional to the mean count [7]. Consequently, reductions in detected photon pairs lead to rapid noise amplification, loss of image contrast, and degradation of structural boundaries. Elevated noise obscures structural delineation and distorts quantitative measurements, particularly for small lesions or at lesion–normal tissue interfaces. Conventional post-reconstruction smoothing or spatial filtering techniques can attenuate high-frequency noise but inevitably compromise spatial resolution and fine structural detail. This trade-off was systematically demonstrated by Arabi, who reported pronounced blurring of small structures and increased quantification bias with Gaussian and bilateral filtering, despite improved signal-to-noise ratio in simulation, phantom, and clinical PET studies [8]. Therefore, reconstructing PET images under low-count conditions represents a fundamental challenge, requiring effective suppression of Poisson-dominated noise while preserving quantitative accuracy and structural fidelity.

To address the inherent limitations of conventional noise-reduction strategies in low-count PET imaging, data-driven image-restoration approaches have been increasingly explored. Deep learning-based methods have demonstrated that they can learn complex, nonlinear mappings between noisy and high-quality images directly from data, enabling effective noise suppression while preserving structural and quantitative information [9,10]. Recent studies have reported that deep learning-based image enhancement improves contrast recovery, edge preservation, and quantitative stability of PET images acquired under reduced-count conditions [11,12,13]. Balaji et al. highlighted that deep learning algorithms consistently benefit PET image enhancement through effective noise reduction, resolution recovery, and improved quantitative performance, while emphasizing the need for rigorous clinical and task-based validation [11]. These advances indicate that deep learning provides a promising framework for overcoming the fundamental trade-offs inherent in low-count PET image reconstruction. Recent studies have demonstrated the expanding role of deep learning frameworks in PET image restoration and enhancement. A comprehensive review by Reader and Corda highlighted the importance of balancing noise suppression and quantitative accuracy in deep learning-based PET reconstruction [14]. Mehranian et al. demonstrated that deep neural networks effectively reduce noise while preserving quantitative fidelity in low-count PET imaging [15]. Weyts et al. further reported that artificial intelligence-based PET denoising enables substantial reduction in acquisition time while maintaining diagnostic image quality [16]. Although these studies demonstrated the effectiveness of deep learning for PET image enhancement, most prior investigations have primarily focused on model implementation and performance comparison. Relatively limited attention has been given to understanding how architectural design factors influence restoration outcomes. Residual learning architectures have been widely adopted due to their optimization stability and strong feature preservation capability. However, the influence of residual network depth—an essential architectural parameter affecting representational capacity, feature reuse, and training stability—remains insufficiently explored in low-count PET image restoration. Among deep learning architectures, convolutional neural networks (CNNs) based on residual learning, such as residual networks (ResNets), have attracted considerable attention for image restoration tasks [17]. Flaus et al. demonstrated that a ResNet-based model significantly outperformed standard PET reconstructions, achieving superior noise suppression and structural preservation, as evidenced by improved peak signal-to-noise ratio and structural similarity metrics, while maintaining quantitative accuracy relative to ground-truth images [18]. This architecture builds on the residual learning principle introduced by He et al., who showed that learning residual mappings, with respect to input features is substantially easier to optimize than learning the underlying mapping. This approach enabled the stable training of very deep networks while preserving essential structural information. Formally, identity-based skip connections in residual networks preserve input information and stabilize gradient propagation during backpropagation, thereby mitigating gradient degradation in deep architectures [19]. Although increasing network depth enhances representational capacity and enables the modeling of more complex degradation patterns, previous studies have shown that excessively deep architectures can introduce training instability and yield diminished performance gains due to optimization difficulties and reduced effective feature utilization [20,21]. Therefore, in low-count PET imaging, where noise is governed by Poisson statistics and structural details are subtle, maintaining an appropriate balance between network depth and reconstruction robustness is critical. However, despite the widespread adoption of residual networks in medical image processing, the influence of the residual network depth on PET image restoration performance under low-count conditions remains insufficiently characterized.

Despite recent advances in deep learning-based PET image restoration, systematic investigation of architectural design factors, particularly residual network depth, remains limited in low-count PET imaging. In addition, previous studies have used datasets acquired under different conditions, which makes differentiation of the effect of network architecture from other influencing factors difficult. The present study aimed to evaluate the impact of residual block depth on PET image restoration under low-count conditions. Low-count PET images reconstructed from 1 min acquisitions were used as input, while 10 min acquisitions served as high-count reference images. By quantitatively comparing networks with different numbers of residual blocks, this study systematically evaluates the influence of residual network depth under controlled acquisition conditions, thereby enabling isolation of architectural effects on low-count PET image restoration and identifying configurations that achieve robust noise suppression while maintaining structural fidelity.

## 2. Materials and Methods

### 2.1. Phantom Experiments

An overview of the experimental workflow is provided as follows. First, PET data were acquired using a controlled 3D-printed striatum phantom under low-count (1 min) and high-count (10 min) conditions. Second, low-count images were used as input data and high-count images as reference targets for model training. Third, ResNet-based models with different residual block depths (8, 16, and 32) were trained for image restoration. Finally, the restored images were quantitatively evaluated using contrast-to-noise ratio (CNR), coefficient of variation (COV), line profile analysis, universal quality index (UQI), and learned perceptual image patch similarity (LPIPS). Phantom experiments were performed using a 3D-printed striatum phantom developed by our research group. The phantom was fabricated from a copper-based filament to provide sufficient gamma ray attenuation for nuclear medicine imaging. The phantom consisted of a cylindrical body containing two symmetric compartments representing the striatal regions. The cylindrical structure had an overall diameter of approximately 15 cm and a height of 4 cm, while the striatal compartments had a vertical dimension of approximately 3 cm. The geometric configuration was designed to mimic the spatial arrangement of the human striatum and to provide controlled and reproducible imaging conditions for evaluating image restoration performance. All images were acquired on a clinical PET/CT system (Biograph Vision 450, Siemens Healthineers, Knoxville, TN, USA). PET data were collected in list mode, allowing retrospective reconstruction with variable acquisition durations. PET images corresponding to both the 1 min and 10 min acquisition durations were reconstructed using an ordered-subset expectation maximization (OSEM) algorithm with time-of-flight information enabled. The reconstruction was performed using eight iterations and five subsets, and resolution recovery based on point-spread function modeling was incorporated. After reconstruction, a Gaussian smoothing filter with a full width at half maximum (FWHM) of 3 mm was applied to suppress high-frequency noise. Attenuation correction was performed using CT data, and additional corrections for scatter, random coincidences, detector dead time, and normalization were applied according to the standard clinical reconstruction protocol. The reconstructed images had a matrix size of 440 × 440 with a voxel size of 1.65 × 1.65 × 1.5 mm^3^.

### 2.2. Modified Median Wiener Filter

To enhance image fidelity in degraded acquisitions, we employed a modified median Wiener filter (MMWF) as a conventional image restoration approach, following the method originally proposed by Cannistraci et al. [22]. The MMWF operates within a Wiener filtering framework, estimating the restored signal from local statistical properties while incorporating a median-based estimator to improve stability against local intensity distortions [23]. The MMWF is defined

(1) $$g\left(n,m\right)=\hat{\mu }+\beta (f\left(n,m\right)-\hat{\mu })$$

(2) $$\beta =\frac{\sigma_{s}^{2}-\sigma_{r}^{2}}{\sigma_{s}^{2}}$$

where $f\left(n,m\right)$ and $g\left(n,m\right)$ represent the degraded input image the restored image at spatial location $\left(n,m\right)$, respectively. The term $\hat{\mu }$ denotes the median intensity within a local neighborhood centered at $\left(n,m\right)$. The parameter $\sigma_{s}^{2}$ corresponds to the local signal variance, and $\sigma_{r}^{2}$ represents the variance associated with image degradation. For comparison, conventional median and Wiener filters were implemented using a 3 × 3-pixel kernel, which is a commonly adopted default configuration for spatial denoising. The MMWF was implemented using a 5 × 5-pixel kernel based on prior studies that demonstrated the effectiveness of this configuration for stable local statistical estimation and noise suppression in medical imaging phantom research [24,25].

### 2.3. Deep Learning Architectures and Hyperparameters

**Figure 1:**
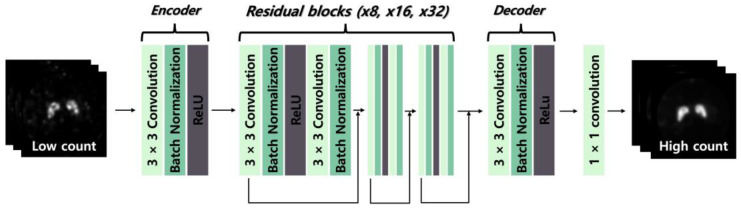
Schematic illustration of the residual network (ResNet)-based image restoration framework with an encoder–residual block–decoder architecture. Low-count positron emission tomography (PET) images are processed through residual blocks with different depths (8, 16, and 32; ResNet-8, ResNet-16, and ResNet-32) to generate high-count PET images.

We designed three ResNet models with 8, 16, and 32 residual blocks (ResNet-8, ResNet-16, and ResNet-32), as illustrated in Figure 1. The general architecture of each model comprises an encoder, a sequence of residual blocks, a decoder, and a final output layer. The encoder extracts key features from the input data through a 3 × 3 convolutional layer, batch normalization, and a rectified linear unit (ReLU) activation function. The proposed network was implemented using 2D convolutional layers and processes single-channel PET images as input to generate single-channel restored PET images as output. We constructed the ResNet model with residual blocks, which were processed in the order of a 3 × 3 convolutional layer, batch normalization, and ReLU activation function, followed by another 3 × 3 convolutional layer and batch normalization for networks containing 8, 16, and 32 residual blocks. All 3 × 3 convolutional layers generate 64 feature maps and employ zero-padding to preserve the spatial dimensions of the feature maps throughout the network. The final restored image is generated using a 1 × 1 convolutional layer. A total of 500 paired PET images were used to construct the dataset. Each sample consisted of a paired input–label image, where the input corresponded to a low-count PET image reconstructed from a 1 min acquisition, and the label corresponded to the matched high-count PET image reconstructed from a 10 min acquisition. These paired images were used for supervised training and evaluation of the deep learning models, enabling direct comparison between restored outputs and corresponding high-count reference images. The paired images were divided into training, validation, and test subsets in a ratio of 8:1:1 (400, 50, and 50 image pairs, respectively) while preserving the input–label correspondence. Each pair was treated as an independent sample during model training and evaluation to ensure non-overlapping data usage between the training and test datasets. The input was a low-count image reconstructed from a 1 min acquisition, while the corresponding 10 min acquisition images were the high-count reference images and used as labels. The learning process was performed using ResNet-based deep learning models with 8, 16, and 32 residual blocks. Model training was conducted using optimized hyperparameters. We used the mean squared error (MSE) as the loss function and the Adam optimizer with a learning rate of 0.0001, a batch size of 10, for 300 epochs. The final hyperparameter configuration was determined through empirical optimization based on preliminary experiments. Learning rates ranging from 1 × 10^−3^ to 1 × 10^−5^, batch sizes between 4 and 16, and training epochs from 100 to 400 were evaluated. The final configuration was selected based on validation performance, with preference given to stable convergence and the lowest validation loss. To ensure reproducibility, data augmentation was limited to random horizontal and vertical flipping. Random seed initialization was fixed for Python (3.13.5), NumPy (2.2.6), and PyTorch (2.7.1) libraries. All models were trained for a predefined number of epochs without early stopping under identical training conditions. Implementation was performed in Python using the PyTorch framework on a workstation with a GeForce RTX 4080 GPU (16 GB of memory). The MSE loss function minimizes pixel-wise intensity differences between output PET images and reference PET images. Mathematically, the proposed method can be interpreted as learning a nonlinear mapping from a low-count PET image to a restored image, where the network parameters are optimized by minimizing the mean squared error between the predicted output and the corresponding high-count reference image during training. Preservation of pixel-level intensity information is important for maintaining quantitative accuracy in PET imaging. Preliminary investigations and prior studies conducted by the research group demonstrated that MSE is the most suitable loss function for preserving quantitative consistency in PET image restoration.

### 2.4. Quantitative Analysis

**Figure 2:**
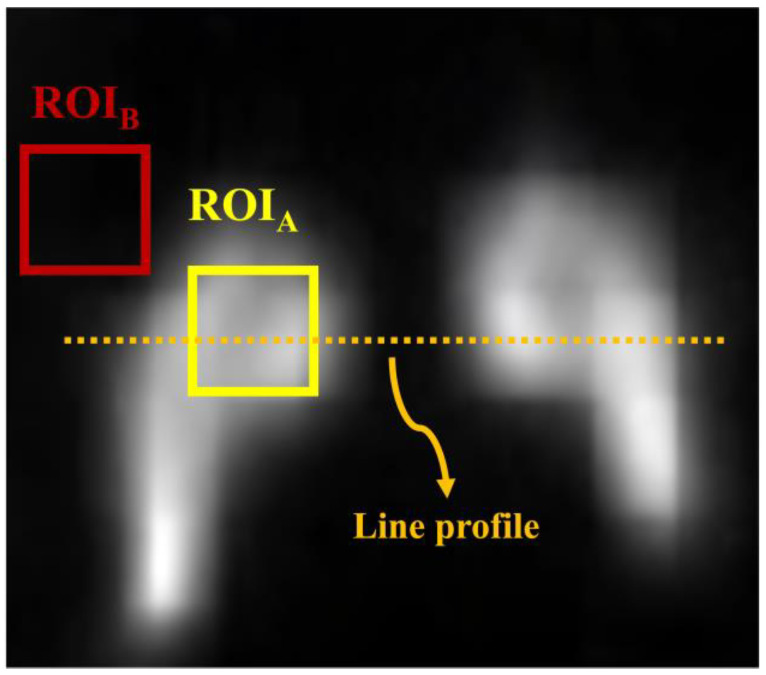
Striatal phantom image with annotations for quantitative analysis. Regions of interest (ROIs) labeled A and B were used to calculate the contrast-to-noise ratio (CNR). ROI_A_ was also used to compute the coefficient of variation (COV).

Image quality was quantitatively evaluated using both conventional and similarity-based metrics. The CNR and COV were calculated from anatomically relevant regions to assess contrast and noise distribution, respectively, as illustrated in Figure 2. Region of interests (ROIs) were defined as fixed square areas positioned over the central region of the striatal compartment to enable quantitative evaluation. ROI placement was standardized by aligning anatomical landmarks to ensure identical spatial coordinates across all images. Each ROI had a size of 11 pixels in width × 11 pixels in height.

Additionally, we assessed image similarity using the UQI and LPIPS to quantify structural consistency and perceptual differences between the images. Line profile analysis was performed to examine local intensity variations across representative structures. The relevant formulae are as follows:

(3) $$CNR=\;\frac{\left|\left.S_{H}-S_{B}\right|\right.}{\sqrt{\sigma_{H}^{2}+\sigma_{B}^{2}}}$$

(4) $$COV=\frac{\sigma_{H}}{S_{H}}$$

where $S_{H}$ and $\sigma_{H}$ denote the mean intensity and standard deviation within the ROIs, respectively, and $S_{B}$ and $\sigma_{B}$ indicate the corresponding mean intensity and standard deviation of the background. In addition, the UQI and LPIPS were calculated as

(5) $$UQI\;(x,\;y)=\frac{4\hat{x}\hat{y}\sigma_{xy}}{\left({\hat{x}}^{2}+{\hat{y}}^{2})(\sigma_{x}^{2}+\sigma_{y}^{2}\right)}$$

where x and y denote the reference image and the evaluated image, respectively. $\hat{x}$ and $\hat{y}$ denote the average intensities of the reference image x and the evaluated image y, respectively. The terms $\sigma_{x}^{2}$ and $\sigma_{y}^{2}$ correspond to the intensity variances of the two images, while $\sigma_{xy}$ represents the cross-covariance between image x and y. The UQI value ranges from −1 to 1, where values closer to 1 indicate higher structural similarity.

(6) $$LPIPS\;\left(x,y\right)=\sum_{l}\frac{1}{H_{l}W_{l}}\sum_{p=1}^{H_{l}}\sum_{q=1}^{W_{l}}{\left\|v_{l}⊙({\hat{F}}_{p,q}^{l}(x)-{\hat{F}}_{p,q}^{l}(y))\right\|}^{2}$$

where ${\hat{F}}_{p,q}^{l}\left(x\right)$ and ${\hat{F}}_{p,q}^{l}(y)$ represent the channel-normalized deep feature vectors extracted from the reference image x and the comparison image y at spatial location (p,q) in the l-th network layer. The spatial resolution of the corresponding feature map is given by $H_{i}\;\times \;W_{i}$ and $V_{l}$ denotes a learned channel-wise weighting vector applied to emphasize perceptually relevant feature differences. In this study, LPIPS was computed using a pre-trained AlexNet-based feature extractor, enabling perceptual dissimilarity to be quantified in a deep feature space rather than through direct pixel-wise comparison. Smaller LPIPS values indicate higher perceptual similarity between images, consistent with human visual perception. Statistical significance testing was not performed because the study was conducted under controlled phantom conditions without inter-patient variability or clinical heterogeneity. The experimental design aimed to evaluate quantitative performance under reproducible physical settings rather than clinical cohort-based statistical inference.

## 3. Results and Discussion

Accurate visualization of striatal structures is clinically important for assessing neurodegenerative disorders such as Parkinson’s disease. However, striatal gamma with PET imaging remains challenging due to the intrinsically low-count nature of nuclear medicine images, which increased noise and obscured fine anatomical detail. Therefore, conventional filtering techniques are commonly used to improve image quality; however, such approaches inevitably involve a trade-off between noise suppression and the preservation of small, clinically relevant structures. Recent advances in deep learning-based image restoration, particularly through residual learning-based CNNs, have shown promising performance in enhancing nuclear medicine images under degraded acquisition conditions. Nevertheless, the impact of a key architectural parameter—network depth, defined by the number of residual blocks—on striatal image restoration has not been systematically evaluated. In this study, we compared conventional filtering methods with ResNet models of varying residual depth using a controlled striatum phantom, investigating the influence of architectural complexity on restoration performance for low-count PET imaging.

**Figure 3:**
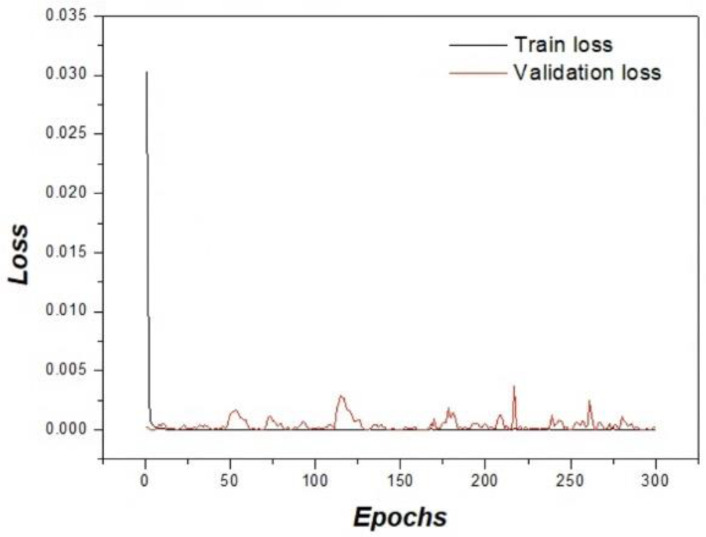
Training and validation loss curves over 300 epochs. The similar trends of both curves indicate stable convergence without significant overfitting.

Figure 3 illustrates the training and validation loss curves across epochs to assess learning stability and potential overfitting. A rapid loss reduction was observed in the early epochs, followed by stable convergence. The close agreement between training and validation curves indicates consistent optimization behavior and suggests that significant overfitting did not occur despite the limited dataset size. Following the validation of training stability, qualitative image restoration performance was further assessed.

**Figure 4:**
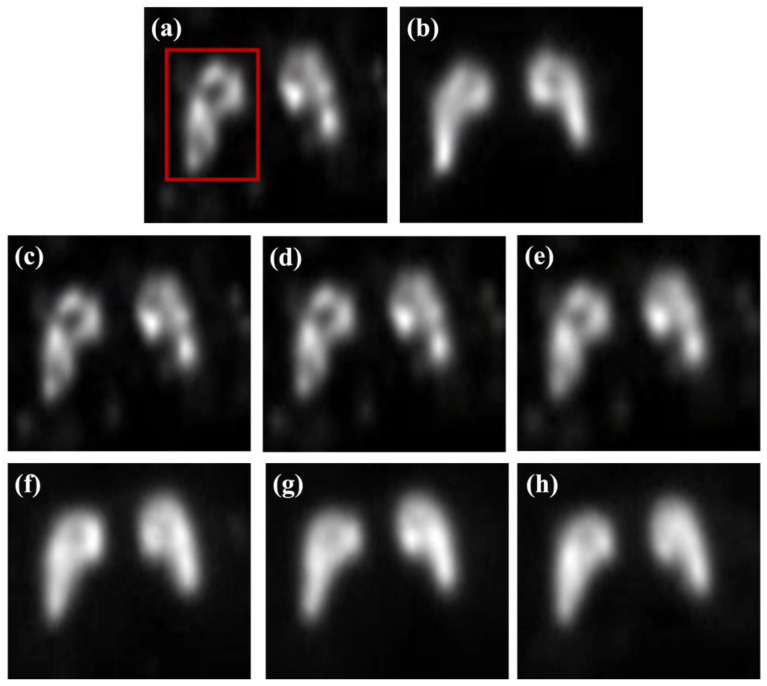
Representative striatum phantom images obtained using various restoration approaches: (**a**) low-count image, (**b**) high-count reference image, (**c**) median filter, (**d**) Wiener filter, (**e**) modified median Wiener filter (MMWF), (**f**) ResNet-8, (**g**) ResNet-16, and (**h**) ResNet-32.

**Figure 5:**
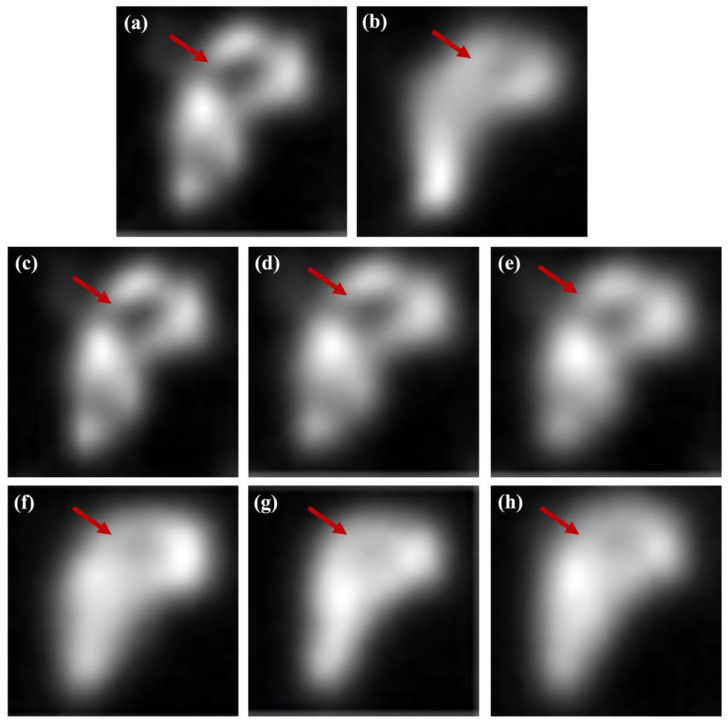
Magnified views of the striatal phantom corresponding to the region of interest (ROI) indicated by the red square in Figure 4: (**a**) low-count image, (**b**) high-count reference image, (**c**) median filter, (**d**) Wiener filter, (**e**) modified median Wiener filter (MMWF), (**f**) ResNet-8, (**g**) ResNet-16, and (**h**) ResNet-32.

Figure 4 presents a qualitative comparison of the striatum phantom images reconstructed using conventional filtering techniques and ResNet-based deep learning models. The low-count images exhibit blurred striatal boundaries and reduced contrast, limiting clear morphological visualization, while the high-count reference images provide improved structural definition. Conventional median filtering effectively suppressed background noise but introduced noticeable smoothing of striatal boundaries. The Wiener filter achieves partial contrast enhancement with limited improvement in boundary delineation. The MMWF demonstrated better preservation of striatal morphology compared to conventional filters, indicating enhanced restoration of structural features through its median-based local estimation. In contrast, ResNet-based models achieved superior restoration performance by learning spatially coherent features beyond local intensity statistics. A progressive improvement in structural delineation was observed with increasing network depth, highlighting the influence of the residual block configuration. As further illustrated in the magnified views in Figure 5, the arrow-marked striatal boundary regions revealed residual blurring in the ResNet-8 model, while the ResNet-16 model provided the clearest delineation of striatal contours with balanced contrast enhancement and structural fidelity. Although the ResNet-32 model achieved comparable contrast recovery, mild over smoothing was observed in localized regions, suggesting that an excessive network depth does not necessarily improve visual quality. Overall, the combined qualitative assessment of Figure 4 and Figure 5 indicates that an intermediate residual block depth, represented by the ResNet-16 architecture, offers the most balanced performance for striatal image restoration under controlled phantom conditions, providing visual support for the subsequent quantitative analyses. Based on these qualitative observations, quantitative evaluation was conducted to further validate the restoration performance.

**Figure 6:**
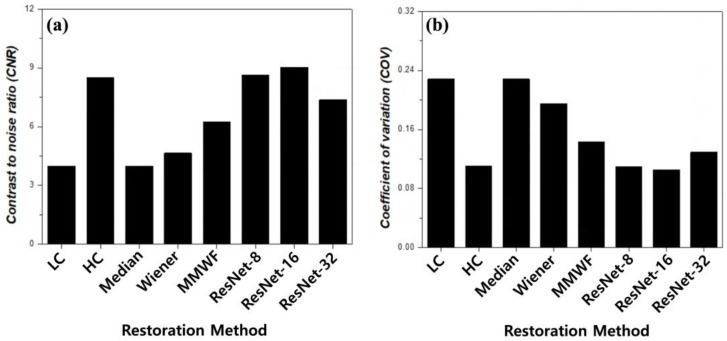
Quantitative comparison of image quality metrics: (**a**) contrast-to-noise ratio (CNR) and (**b**) coefficient of variation (COV) for different restoration methods. LC and HC denote low-count and high-count images, respectively, and MMWF indicates modified median Wiener filter. Higher CNR values indicate improved contrast, whereas lower COV values indicate enhanced signal stability.

Figure 6 and Table 1 present a quantitative evaluation of striatal image quality using the CNR and COV. Low-count PET imaging is intrinsically affected by Poisson-dominated statistical noise arising from insufficient detected photon events. This stochastic noise increases voxel-wise intensity fluctuations and reduces peak-to-background separability, leading to degraded contrast and unstable signal distribution. As a result, quantitative metrics sensitive to signal variation—such as contrast-to-noise ratio and coefficient of variation—are directly influenced by the noise level. Therefore, in this study, restoration performance was systematically assessed using both qualitative visualization and quantitative metrics to evaluate the effectiveness of each method in suppressing noise-induced contrast degradation and signal instability. The low-count input image exhibited low contrast (CNR = 3.99) and high intensity variability (COV = 0.228). In contrast, the high-count reference image provided substantially improved contrast (CNR = 8.92) and reduced variability (COV = 0.111), establishing a benchmark for optimal quality. Conventional filtering techniques showed limited contrast restoration capability. The median filter produced no appreciable CNR improvement compared to the low-count input, while the Wiener filter yielded a modest increase to 4.65. The MMWF achieved a CNR of 6.25, corresponding to a 1.57-fold increase relative to the input. Despite this improvement, the contrast recovery from conventional and hybrid filtering remained inferior to the deep learning-based approaches. However, the ResNet-based models demonstrated substantially higher CNR values. The ResNet-8 model achieved a CNR of 8.64 (a 2.17-fold increase). The ResNet-16 model yielded the highest CNR of 9.02 (a 2.26-fold improvement), which slightly exceeded the high-count reference level (1.01-fold). In contrast, the ResNet-32 model produced a lower CNR of 7.37, suggesting diminishing returns on contrast recovery with excessive network depth. In low-count PET imaging, where image degradation is predominantly governed by Poisson noise and preservation of subtle anatomical structures is essential, balanced feature representation becomes more important than excessive network depth [26]. Signal stability analysis based on COV revealed complementary trends. The median filter maintained a high-intensity variability comparable to the input. The Wiener filter reduced the variability to 0.195; additionally, the MMWF achieved a COV of 0.143. Deep learning-based approaches provided the lowest overall variability. The ResNet-8 model yielded a COV of 0.110, closely matching the high-count reference. The ResNet-16 model achieved the lowest COV of 0.105. The ResNet-32 model exhibits increased variability (COV = 0.129), indicating reduced signal stability with excessive network depth. Together, the separated CNR and COV analyses demonstrated that an intermediate residual block configuration provided the most favorable balance between contrast enhancement and signal stability. The ResNet-16 architecture achieved the highest contrast recovery while simultaneously minimizing intensity variability, quantitatively supporting the qualitative observations in Figure 4 and Figure 5. These numerical results confirmed that increasing the network depth beyond an intermediate level yields no additional benefits for striatal image restoration under these controlled phantom conditions. Overall, the ResNet-16 model provided the most balanced performance, achieving the highest CNR and lowest COV among all evaluated methods.

**Figure 7:**
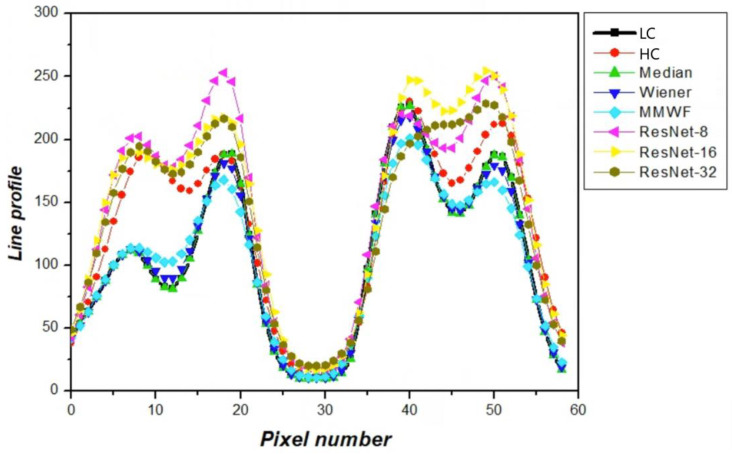
Line profile analysis showing intensity variations along the selected line across the striatum region for different restoration methods. The profiles allow comparison of peak intensity and contrast recovery, reflecting the ability of each method to preserve structural details.

Figure 7 presents a line profile analysis of the striatum phantom images, evaluating the local intensity variation and structural continuity across the different restoration methods. This analysis provides information complementary to region-based metrics by directly examining peak amplitude, valley depth, and signal transitions along a predefined spatial trajectory. The low-count input image exhibited attenuated peak-to-valley contrast and flattened signal transitions, resulting in limited separation between the bilateral striatal structures. Conventional filtering techniques partially recovered peak amplitude but introduced profile smoothing, leading to reduced valley depth and diminished structural distinction. The MMWF demonstrated improved peak restoration and valley preservation compared to standard filters; however, its overall profile amplitude remained lower than that of the high-count reference. The ResNet-based models showed substantial improvements in preserving the characteristic shape of the striatal intensity profile. The ResNet-8 model enhanced peak amplitude and improved separation between striatal regions but retained residual smoothing in transition regions. The ResNet-16 model exhibited the closest agreement with the high-count reference profile, characterized by well-defined peak positions, preserved valley depths, and smooth yet distinct signal transitions. The ResNet-32 model produced an elevated peak amplitude; however, partial filling of valley regions and reduced profile sharpness were observed, suggesting excessive smoothing or over-regularization. From a mechanistic perspective, network depth influences restoration performance through its impact on representational capacity, feature utilization efficiency, and optimization stability. Shallow networks such as ResNet-8 provide limited hierarchical feature abstraction, which constrains their ability to model complex Poisson noise characteristics and spatial intensity variations, resulting in reduced structural fidelity and quantitative similarity. In contrast, intermediate-depth architectures achieve a balanced level of feature abstraction and stable gradient propagation, enabling effective recovery of structural boundaries while maintaining intensity consistency. However, excessively deep networks may introduce optimization instability and redundant feature propagation, leading to over-smoothing and diminished structural detail. These effects are particularly pronounced in low-count PET imaging, where subtle anatomical features require efficient feature representation rather than excessive architectural complexity.

**Figure 8:**
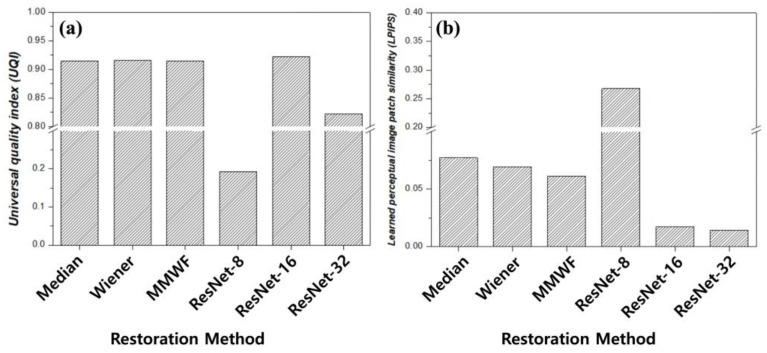
Quantitative similarity analysis between the high-count (HC) reference image and images obtained using different restoration methods: (**a**) universal quality index (UQI) and (**b**) learned perceptual image patch similarity (LPIPS). Higher UQI values indicate greater structural similarity, whereas lower LPIPS values indicate improved perceptual similarity. The horizontal line indicates a break in the y-axis scale.

Figure 8 and Table 2 present a quantitative similarity analysis using the UQI and LPIPS to evaluate the structural and perceptual similarity between the restored and the high-count reference images. UQI values closer to unity indicate higher structural similarity, while lower LPIPS values indicate greater perceptual similarity to the reference. Conventional filtering techniques exhibited relatively high UQI values, ranging from 0.9146 to 0.9158, indicating the preservation of global structural similarity. However, the corresponding LPIPS values remained elevated: 0.0772 for the median filter, 0.0695 for the Wiener filter, and 0.0614 for the MMWF. This disparity suggests that conventional filtering approaches maintain coarse structural agreement but fail to recover the fine perceptual features of the reference image. These results indicate that conventional filters primarily preserve coarse structural information while failing to recover the fine perceptual features present in high-count images. In contrast, the ResNet-based models demonstrated a more pronounced trade-off between structural and perceptual metrics. The ResNet-8 model exhibits a substantially reduced UQI value of 0.1925 and a high LPIPS of 0.2681, indicating poor agreement with the reference image in both domains. In addition, the relatively shallow residual structure of ResNet-8 limits its representational capacity to model complex noise characteristics and intensity distributions. This limitation reduces global structural consistency, leading to lower UQI values. In addition, restricted feature abstraction weakens texture and detail reconstruction, resulting in increased perceptual dissimilarity and higher LPIPS scores. These findings consistently indicate reduced restoration accuracy compared with deeper residual networks. In contrast, the ResNet-16 model achieved the highest UQI (0.9224) among all methods and a markedly reduced LPIPS (0.0174), indicating superior preservation of structural integrity and perceptual similarity. The ResNet-32 model yielded a lower UQI (0.8222) but the lowest LPIPS (0.0142), suggests diminished structural fidelity despite enhanced perceptual similarity. The integrated interpretation of the UQI and LPIPS demonstrates that optimal image restoration requires the simultaneous preservation of structural information and perceptual realism. The ResNet-16 architecture demonstrates a relatively balanced performance by maintaining high structural similarity while achieving substantial perceptual alignment. The quantitative similarity analysis supports the qualitative observations presented in Figure 4 and Figure 5, as well as the line-profile assessment shown in Figure 7. Collectively, these results suggest that an intermediate residual block depth provides improved restoration performance for striatum phantom imaging under the present controlled conditions. The results of the present study are consistent with previous reports demonstrating the effectiveness of deep learning-based PET denoising methods. Hashimoto et al. developed a personalized deep learning strategy for low-count PET imaging and reported that deep learning models can effectively suppress noise while preserving image quality without increasing tracer dose or scan time [27]. Furthermore, Liu et al. summarized recent developments in deep learning-based PET reconstruction and highlighted that data-driven approaches significantly improve image quality under low-count conditions by learning the mapping between noisy and high-quality PET data [28]. These findings are in agreement with the present results, where residual learning-based architectures improved image quality by reducing noise and preserving structural information in low-count PET images. Overall, the results consistently demonstrate that an intermediate residual block depth yields optimal restoration performance, supporting the effectiveness of the ResNet-16 configuration under the present conditions.

Across all experimental conditions, the proposed method consistently improved both quantitative metrics (e.g., CNR and COV) and perceptual quality indicators (e.g., UQI and LPIPS) compared with conventional filtering approaches, indicating stable performance under controlled variations. Although the current study was conducted under controlled phantom conditions, the consistent improvement observed across multiple configurations suggests the potential robustness of the proposed approach beyond the specific experimental setting. Despite the promising findings, this study has several limitations. First, the evaluation was conducted using a controlled physical striatum phantom. While this enabled a reproducible and objective assessment of image restoration performance, it could not fully capture the biological variability, complex tracer kinetics, and motion artifacts present in clinical imaging [29]. Consequently, the generalizability of the observed optimal residual block configuration to patient data requires validation. Second, the analysis focused on a specific residual network architecture and a limited range of depths; therefore, the potential influence of other architectural factors, such as different backbone designs, attention mechanisms, and loss functions, remains unexplored. In addition, the dataset size was relatively limited, which may constrain statistical reliability and reduce the generalizability of the results to broader imaging conditions [30]. The quantitative evaluation was based on representative values obtained under controlled phantom conditions. Because repeated independent measurements were not available, formal statistical significance testing could not be performed. Future studies will incorporate repeated measurements and larger datasets to enable rigorous statistical analysis. Furthermore, the performance of the proposed method on arbitrary clinical images and in the presence of pathological variations remains to be investigated. Future studies will extend this framework to clinical PET and SPECT datasets to assess its robustness under realistic imaging conditions and to investigate the interaction between network depth and other architectural components. These investigations are essential for establishing practical design guidelines for deep learning-based restoration models that effectively balance image quality enhancement with structural fidelity in clinically relevant tasks. These limitations may affect the generalizability of the present findings, particularly when applied to clinical scenarios involving patient-specific variability, motion, and heterogeneous tracer distribution. Therefore, careful interpretation is required when extending the current results to real-world clinical PET imaging.

## 4. Conclusions

This study evaluated PET image restoration under low-count conditions using a physically controlled striatum phantom, with a focus on the influence of residual block depth in ResNet-based architectures. Conventional filtering techniques show limited improvement in contrast recovery and signal stability, while residual learning-based models provided superior restoration by preserving structural boundaries and reducing intensity variability. Quantitative analysis using CNR, COV, line profiles, and similarity metrics demonstrated that residual block configuration critically affects restoration performance. Among the evaluated models, the ResNet-16 architecture achieved the most balanced performance, providing enhanced contrast recovery, stable signal uniformity, and high structural and perceptual similarities to the high-count reference images. These results indicate that an intermediate residual block depth offers optimal restoration capability for striatal PET imaging under controlled conditions and highlights the importance of architectural optimization in deep learning-based PET image enhancement. The present findings were derived from controlled phantom experiments designed for reproducible performance evaluation. Future studies will extend this framework to clinical patient datasets to evaluate its practical applicability under real-world imaging conditions. Statistical validation will also be performed in clinical evaluations to confirm the generalizability and robustness of the proposed approach.

## Figures and Tables

**Table 1:** Quantitative evaluation of restoration performance under low-count positron emission tomography (PET) conditions using contrast-to-noise ratio (CNR) and coefficient of variation (COV) across conventional filtering methods and residual network (ResNet) models. LC and HC denote low-count and high-count images, respectively, and MMWF indicates modified median Wiener filter. Higher CNR values indicate improved contrast, whereas lower COV values indicate enhanced signal stability.

	LC	HC	Median	Wiener	MMWF	ResNet-8	ResNet-16	ResNet-32
CNR	3.99	8.92	3.99	4.65	6.25	8.64	9.02	7.37
COV	0.228	0.111	0.228	0.195	0.143	0.110	0.105	0.130

**Table 2:** Quantitative comparison of similarity metrics, including universal quality index (UQI) and learned perceptual image patch similarity (LPIPS), across conventional filtering methods and residual network (ResNet) models. MMWF indicates modified median Wiener filter. Higher UQI values indicate greater structural similarity to the reference image, whereas lower LPIPS values indicate improved perceptual similarity.

	Median	Wiener	MMWF	ResNet-8	ResNet-16	ResNet-32
UQI	0.9146	0.9158	0.9150	0.1925	0.9224	0.8222
LPIPS	0.0772	0.0695	0.0614	0.2681	0.0174	0.0142

## Data Availability

The datasets generated and/or analyzed in the current study are available from the corresponding author upon reasonable request.
